# A Novel Regulatory Circuit “C/EBPα/miR-20a-5p/TOB2” Regulates Adipogenesis and Lipogenesis

**DOI:** 10.3389/fendo.2019.00894

**Published:** 2020-01-08

**Authors:** Jie Zhou, Junying Yang, Xiaochen Wang, Mengyue Li, Fang Li, Endong Zhu, Xuemei Li, Xiaoxia Li, Baoli Wang

**Affiliations:** ^1^NHC Key Lab of Hormones and Development, Tianjin Key Lab of Metabolic Diseases, Chu Hsien-I Memorial Hospital & Institute of Endocrinology, Tianjin Medical University, Tianjin, China; ^2^Department of Microbiology, College of Basic Medical Sciences, Tianjin Medical University, Tianjin, China

**Keywords:** microRNA, adipocyte differentiation, lipogenesis, TOB2, C/EBPα

## Abstract

Recent studies have identified growing importance of microRNAs as key regulators of adipocyte differentiation. We have previously reported that miR-20a-5p is able to induce adipogenesis of established adipogenic cell lines and bone marrow derived mesenchymal stem cells (BMSCs). However, the molecular mechanisms by which miR-20a-5p controls adipogenesis and by which miR-20a-5p expression is regulated need to be further explored. In the current study we found that miR-20a-5p expression was induced during adipocyte differentiation from preadipocyte 3T3-L1 and was increased in epididymal white adipose tissue from either ob/ob mice or high fat diet-induced obese mice. Functional studies identified miR-20a-5p as a positive regulator of adipocyte differentiation and lipogenesis in 3T3-L1 by using either synthetic mimics to supplement miR-20a-5p, or using synthetic inhibitor or sponge lentivirus to inactivate endogenous miR-20a-5p. Luciferase activity assay revealed that TOB2 is a novel target of miR-20a-5p and functional experiment demonstrated its negative regulatory role in adipocyte differentiation. Moreover, Tob2 overexpression significantly attenuated adipocyte formation induced by miR-20a-5p supplementation. In-depth investigation of mechanisms that govern miR-20a-5p expression clarified that C/EBPα transcriptionally activated miR-20a-5p expression via binding to the promoter of miR-20a-5p. Taken together, we conclude that a novel C/EBPα/miR-20a-5p/TOB2 circuit exists and regulates adipogenesis and lipogenesis.

## Introduction

Obesity is a widespread health problem, characterized by excessive body fat mass, and is strongly correlated with health disorders such as cardiovascular disease, hypertension, diabetes mellitus type 2, and metabolic syndrome (1). Fat accumulation primarily depends on the increasing number (hyperplasia) and/or size of adipocytes (hypertrophy) (2). Adipocytes are regarded as one of the major targets in the prevention and treatment of obesity and related metabolic syndrome (3). Thus, it is an urgent task to understand the molecular genetic mechanisms involved in adipogenesis.

Adipocyte differentiation is a program regulated by a cascade of multiple transcription factors, among which two major transcription factors, i.e., peroxisome proliferator-activated receptor γ (PPARγ) and CCAAT/enhancer-binding protein α (C/EBPα) are of critical importance (4–7). C/EBPα and PPARγ positively regulate each other and mediate the activation of a variety of downstream targets, such as adipocyte protein 2 (aP2) (8). Besides, sterol regulatory element binding protein 1 (SREBP1) also plays an important role in adipocyte differentiation and lipogenesis through modulating some key lipogenic enzymes including acetyl-CoA carboxylase 1 (ACC1) and fatty acid synthase (FASN), leading to synthesis and storage of lipid droplets (9).

microRNAs (miRNAs) function as key regulators of gene expression in a variety of metabolism-regulatory tissues and cell types including adipocytes (10, 11). Different microRNAs may either promote or inhibit adipogenesis through targeting different downstream signals (12–16). Moreover, some microRNAs have been implicated in diet-induced obesity. Data from Price et al. showed that miR-33 null mice had increased food intake, enhanced adipose tissue expansion, and a predisposition to develop diet-induced obesity and insulin resistance (17). miR-143 was found to be upregulated in mesenteric fat of high-fat diet-induced mice (18).

miR-20a-5p, as a member of the miR-17-92 gene cluster, has critical roles in tumorigenesis and development (19, 20). We have recently demonstrated that miR-20a-5p regulates adipogenesis in preadipocytes as well as marrow stromal cells (21, 22). However, the mechanism underlying the function of miR-20a-5p in governing adipogenesis, as well as the mechanism how miR-20a-5p is regulated, has not yet been fully elucidated.

In this research, we have characterized miR-20a-5p to be a downstream transcriptional target of C/EBPα and further investigated its role in adipocyte differentiation and lipogenesis in preadipocyte 3T3-L1. We have for the first time demonstrated that transducer of ERBB2, 2 (Tob2) is a novel target of miR-20a-5p and negatively regulates adipogenesis and lipogenesis. We propose a regulatory machinery in which C/EBPα/miR-20a-5p/TOB2 circuit controls the cell fate of preadipocytes.

## Materials and Methods

### Cell Cultures

3T3-L1 preadipocytes were grown in Dulbecco's modified Eagle medium (DMEM, GIBCO) containing 10% Fetal Bovine Serum (FBS, Invitrogen, Carlsbad, CA, USA). For adipogenic differentiation, the cells at 100% confluence were induced for 3 days to allow adipocyte differentiation in the medium of α-MEM containing 10% FBS, 0.5 μM dexamethasone, 5 μg/ml insulin, 50 μM indomethacin, and 0.25 mM methylisobutylxanthine. Then the cells were cultured in the presence of 5 μg/ml insulin alone for 2 more days.

Bone marrow stromal cells (BMSCs) were isolated from femurs and tibias of 3-month-old mice and cultured to induce adipocyte differentiation as previously described (23).

### RNA Extraction and Quantitative RT-PCR

Total RNA was extracted with a total RNA isolation kit (Omega Bio-Tek, Norcross, GA, USA). First-strand cDNA synthesis and PCR amplifications were performed following a previously reported protocol (21). The relative expression levels of the target genes were determined by the comparative Ct (ΔΔCt) method using β-actin as the internal control.

For miRNA analysis, RNA was isolated using a miRNA extraction kit (Omega Bio-Tek, Norcross, GA, USA), and reverse-transcribed into cDNA using the stem-loop RT primer, 5′-GTCGTATCCAGTGCAGGGTCCGAGGTATTCGCACTGGATACGACCTACCTGCACTATA-3′. Quantitative PCR amplifications were done using a SYBR Green master mix (Sangon, Shanghai, China). The cycling scheme included 40 cycles of denaturation at 95°C for 10 s, annealing at 60°C for 10 s, and extension at 72°C for 10 s. U6 was employed as internal control. The PCR primers used are listed in Table S1.

### Luciferase Reporter Assays

The prediction of target mRNAs was done online (http://www.targetscan.org/). We PCR-amplified the fragment of Tob2 3′-UTR carrying the putative miR-20a-5p binding sequence with cDNA of 3T3-L1 cells as the template. The PCR fragment was subcloned into pMir-report vector (Invitrogen) at SacI/HindIII restriction sites using the ClonExpress II One Step Cloning Kit (Vazyme, Nanjing, China). Each transfection was done in HEK-293 cells with the miR-20a-5p mimics or the negative control, the Tob2 3′-UTR construct, along with Renilla luciferase reporter vector pRL-SV40 using Attractene Transfection Reagent (Qiagen, USA). Thirty-six hours after transfection, the cells were harvested and subjected to luciferase assays using a dual-luciferase reporter assay kit (Promega, San Luis Obispo, CA, USA). The relative luciferase activity was determined by dividing the firefly luciferase activity with Renilla luciferase activity.

For the miR-20a-5p promoter study, the mouse miR-20a-5p promoter fragment (1.2 kb, −1,240~ +3) was generated by PCR using specific primers (Table S1). The product was then subcloned into the vector pGL3-basic (Promega, San Luis, CA, USA) at the restriction sites of XhoI and HindIII. PCR-based point mutations to the three potential C/EBPα binding sequences were made by using a kit (Vazyme, Nanjing, China) with the wild-type promoter construct as the template. The potential binding sequences of C/EBPα were mutated to 11-mer dT. To verify the role of C/EBPα in miR-20a-5p transcription, 3T3-L1 cells were transfected with the mutant or the wild-type constructs of miR-20a-5p promoter and C/ebpα siRNA (Jima Biotech, Shanghai, China), or the negative control siRNA using Lipofectamine 3000 (Invitrogen). The sequences of C/ebpα siRNA are as follows: sense, 5′-GCUUUAUCAUCCGAUAUCAACACTT-3′; antisense, 5′- AAGUGUUGAUAUCGG AUGAUAAAGCAA-3′. pRL-SV40 was also included to monitor the efficiency of the transfection. The luciferase activity was measured 36 h after transfection.

### Transfection of miRNA Mimics, Inhibitor, and Constructs

The mimics and inhibitor of miR-20a-5p and their negative controls were synthesized by Genepharma (Shanghai, China). These small molecules were transfected for 16 h into 3T3-L1 cultures using lipofectamine RNAi-max (Invitrogen), respectively. The mimics and inhibitor was used at final concentration of 50 and 100 nM, respectively. When reaching 100% confluence, the cultures were induced with adipogenic medium to allow differentiation.

To investigate the involvement of TOB2 in the function of mir-20a-5p, Tob2 construct was made by incorporating the translated region of mouse Tob2 into pcDNA3.1(+) vector at the restriction sites of BamHI and EcoRI. Tob2 construct or the vector was singly transfected or cotransfected with the miR-20a-5p mimics or the negative control into 3T3-L1 cultured in a 24-well plate using Attractene Transfection Reagent. 16 h after transfection, the culture medium was refreshed. Adipogenic agents were added to the confluent cultures to allow differentiation.

### Lentiviral Packaging and Infection

Sponge oligos containing 6 tandem antisense sequences of miR-20a-5p were synthesized and cloned into the shRNA lentiviral transfer vector pLVX-shRNA2 (Clontech, Palo Alto, CA, USA). The lentiviruses expressing miR-20a-5p sponge or the empty vector were packaged as previously described (21). The 3T3-L1 cells were infected with the lentiviruses at an MOI (multiplicity of infection) of 10. Then, the cells of 100% confluence were allowed to differentiate in presence of adipogenic medium.

### Oil-Red O Staining and Triglyceride Assay

Differentiated adipocytes were washed with phosphate-buffered saline (PBS), then fixed for 10 min in 4% paraformaldehyde. After washing with deionized water, we stained the cells for 5 min with 0.3% oil-red O in 60% saturated isopropanol.

To quantify the lipid storage of the cells, triglyceride content was measured using the Triglyceride GPO-PAP kit (Jiancheng Biotechologies, Nanjing, China). The absorbance was measured at 510 nm, and triglyceride was normalized to the total protein concentration measured by a BCA assay kit (Biomed Biotechologies, Beijing, China).

### Chromatin Immunoprecipitation Assay

Chromatin immunoprecipitation (ChIP) experiment was done using a kit from Cell signaling Technology (Danvers, MA, USA). Cell lysates containing soluble chromatin were incubated overnight with 4 μg rabbit anti-C/EBPα antibody (Proteintech, Wuhan, China) or rabbit IgG. The DNA was de-cross-linked, then subjected to PCR amplification of mouse miR-20a-5p promoter-specific sequences. The primers are listed in Table S1.

### Western Blotting

Western blotting experiments were performed as previously described (23). The primary antibodies used are: rabbit monoclonal or polyclonal antibodies by Cell Signaling Technology: anti-PPARγ (#2443, 1:1000), anti-Perilipin (#9349, 1:1000), anti-C/EBPα (#8178, 1:1000) and anti-ACC1 (#4190, 1:1000); rabbit monoclonal or polyclonal antibodies by Proteintech (Wuhan, China): anti-aP2 (12802-1-AP, 1:1000), anti-FASN (10624-2-AP, 1:1000), anti-SREBP1 (66875-1-Ig, 1:2000) and anti-β-actin (66009-1-Ig, 1:5000); rabbit polyclonal antibody by Abclonal (Wuhan, China): anti-TOB2 (A7223, 1:1000). Protein bands were visualized using a chemiluminescence reagent (Advansta, Menlo Park, CA) and analyzed with Image J software.

### Mice

Three-month-old ob/ob mice and their WT litternates were purchased from Biomedical Research Institute of Nanjing University (Nanjing, China). Healthy C57BL6/J mice were purchased from Hua Fu Kang Biological Technology (Beijing, China) and fed with normal-fat diet (NFD), or high-fat diet (HFD) for 8 weeks as previously described (24). The animal experiments were done in accordance with the Chinese guidelines for animal welfare and experimental protocol, and was approved by the Animal Ethics Committee of Tianjin Medical University.

### Statistics Analysis

The results are represented as means ± SD. Independent *t*-test or one-way ANOVA was performed after the test of homogeneity of variances. If the one-way ANOVA indicates significant difference, the Student-Newman-Keuls test was further done to make a *post-hoc* comparison. The difference was considered statistically significant if *p* < 0.05.

## Results

### miR-20a-5p Was Upregulated During Adipocyte Differentiation and in the White Adipose Tissue of Obese Mice

**Figure 1:**
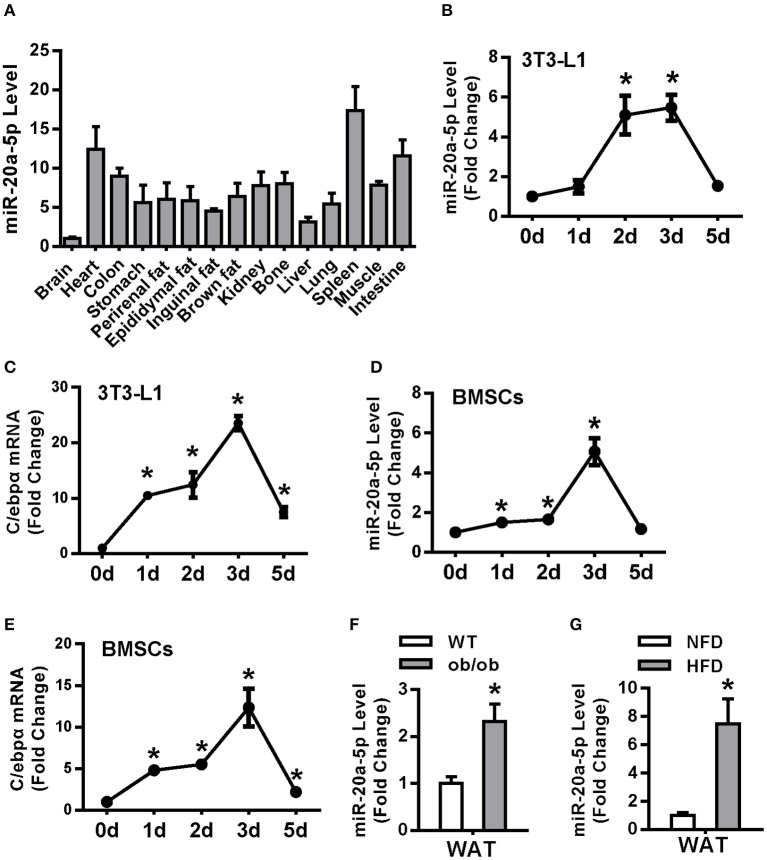
miR-20a-5p was upregulated during adipocyte differentiation and in the WAT of obese mice. **(A)** miR-20a-5p expression in various tissues of mice was analyzed by qRT-PCR. The level of miR-20a-5p in brain was set at 1. **(B–E)** Expression levels of miR-20a-5p and C/EBPα during adipogenesis of 3T3-L1 preadipocytes **(B,C)** or BMSC **(D,E)** were analyzed by qRT-PCR. **(F,G)** miR-20a-5p level was analyzed in epididymal WAT of ob/ob mice and HFD-fed mice. The levels of the measurements at day 0 were set as 1 in **(B–E)**. Values are the means ± SD, *n* = 3 in **(B–F)**, *n* = 5 in **(G)**. *Significant vs. day 0 **(B–E)** or WT **(F)** or NFD **(G)**, *p* < 0.05.

miR-20a-5p was expressed in various tissues in 9-week-old-mice and its level was high in spleen, heart, intestine and colon, moderate in kidney, bone and various white fat and interscapular brown fat tissues (Figure 1A). miR-20a-5p and C/ebpα mRNA were synchronously increased at day 2 and 3 during adipogenesis of 3T3-L1 (Figures 1B,C). In primary cultured BMSCs, the expression patterns of miR-20a-5p and C/ebpα were similar to those in 3T3-L1 (Figures 1D,E). Moreover, miR-20a-5p level was induced in epididymal white adipose tissue (WAT) of ob/ob mice and HFD-fed obese mice as compared with non-obese controls (Figures 1F,G). By contrast, it was reduced in interscapular brown adipose tissue (BAT) of ob/ob mice and HFD-fed mice (Figure S1). These data suggest a potential role of miR-20a-5p in adipocyte differentiation and obesity.

### miR-20a-5p Regulated Adipocyte Differentiation and Lipogenesis of 3T3-L1 Cells Mainly at the Early Stage

**Figure 2:**
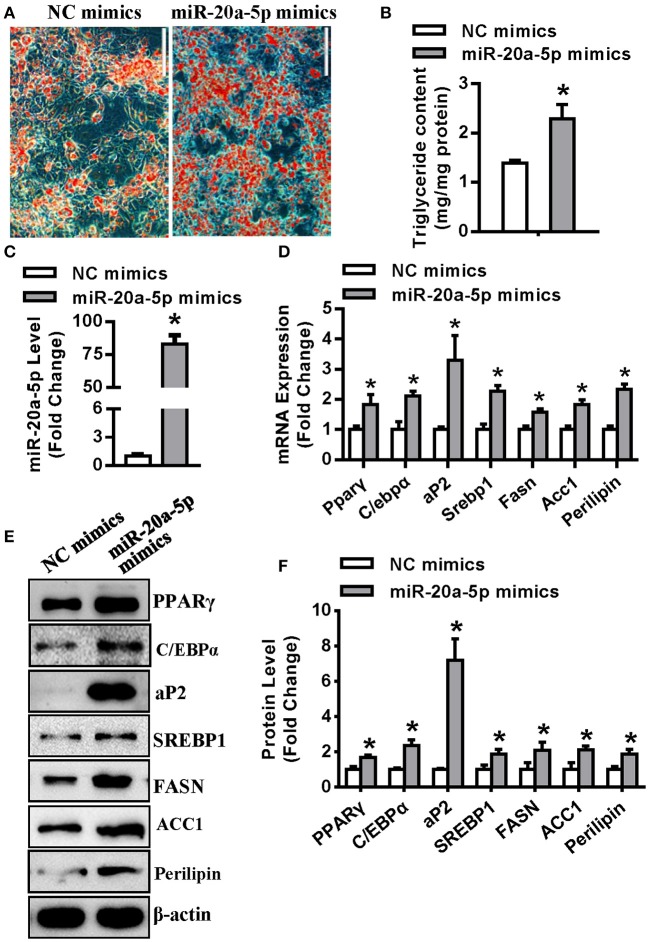
Supplementing miR-20a-5p promoted adipogenesis and lipogenesis. **(A,B)** Differentiated adipocytes were stained with oil-red O and intracellular triglyceride contents were measured 5 days after adipogenic induction. **(C)** qRT-PCR verified the level of miR-20a-5p in 3T3-L1 3 days after adipogenic induction. The mRNA **(D)** and protein **(E)** levels of adipogenic and lipogenic factors were analyzed by qRT-PCR and Western blotting. **(F)** Protein expression levels were quantified. Scale in **(B)** 200 μm. Values are the means ± SD (*n* = 3). *Significant vs. negative control (NC), *p* < 0.05.

Transfection of miR-20a-5p mimics to undifferentiated 3T3-L1 resulted in efficient conversion to mature adipocytes, with significant increase in triglyceride content (Figures 2A,B). The level of miR-20a-5p was increased by 83-fold in 3T3-L1 cells 3 days after adipogenic induction (Figure 2C). 72 h following adipogenic treatment, the mRNA levels of adipogenic factors Pparγ, C/ebpα and aP2 were 1.8-, 2.1-, and 3.2-fold, respectively, greater in cells supplementing miR-20a-5p mimics than in those transfected with negative control (NC) mimics. Similarly, lipogenic factors including Srebp1, Fasn, Acc1 and lipid droplet-containing protein Perilipin were increased by 2.2-, 1.5-, 1.8-, and 2.3-fold, respectively, in cells supplementing miR-20a-5p mimics vs. control cells (Figure 2D). Consistently, miR-20a-5p mimics increased the protein levels of these adipogenic and lipogenic factors 72 h after adipogenic induction (Figures 2E,F).

Transfections were also carried out in differentiated 3T3-L1 under adipogenic treatment for 3 days. Two days after transfection, qRT-PCR revealed a 80-fold increase of miR-20a-5p level in the cells transfected with miR-20a-5p mimics vs. those with control mimics, demonstrating the efficacy of transfection (Figure S2A). However, the supplementation of miR-20a-5p failed to further enhance adipocyte differentiation and to potentiate the mRNA and protein expression of the adipogenic factors (Figures S2B–F).

**Figure 3:**
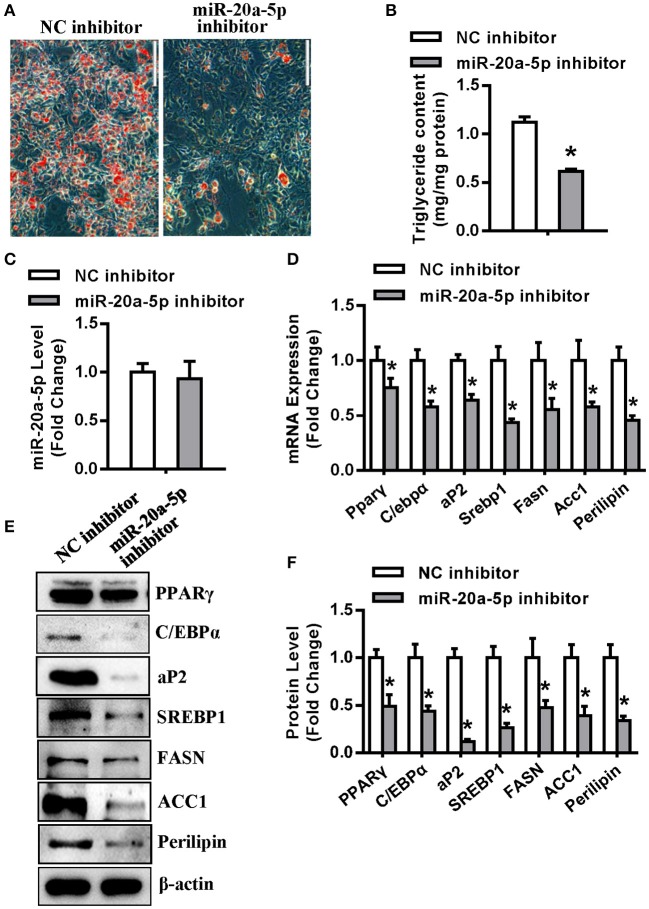
Inhibition of endogenous miR-20a-5p suppressed adipogenesis and lipogenesis. **(A,B)** Differentiated adipocytes were stained with oil-red O and intracellular triglyceride contents were measured 5 days after adipogenic induction. **(C)** qRT-PCR detected the level of miR-20a-5p in 3T3-L1 3 days after adipogenic induction. The mRNA **(D)** and protein **(E)** levels of adipogenic and lipogenic factors were analyzed by qRT-PCR and Western blotting. **(F)** Protein expression levels were quantified. Scale in **(A)** 200 μm. Values are the means ± SD (*n* = 3). *Significant vs. NC, *p* < 0.05.

In contrast, transfection of miR-20a-5p inhibitor into undifferentiated 3T3-L1 reduced the formation of mature adipocytes (Figure 3A), along with significant decrease in triglyceride content (Figure 3B). Three days after adipogenic induction, the level of miR-20a-5p did not significantly change in the cells transfected with miR-20a-5p inhibitor (Figure 3C). The mRNA and protein expression of adipogenic and lipogenic factors was significantly declined in cells transfected with miR-20a-5p inhibitor vs. those transfected with NC 72 h after adipogenic treatment (Figures 3D–F).

Transfections of miR-20a-5p inhibitor were also carried out in differentiated 3T3-L1 under adipogenic treatment for 3 days. The inhibition of miR-20a-5p did not alter adipocyte differentiation and attenuate the mRNA and protein expression of the adipogenic and lipogenic factors (Figures S3A–E). The data suggested that miR-20a-5p promotes adipogenesis mainly at early stage of differentiation.

**Figure 4:**
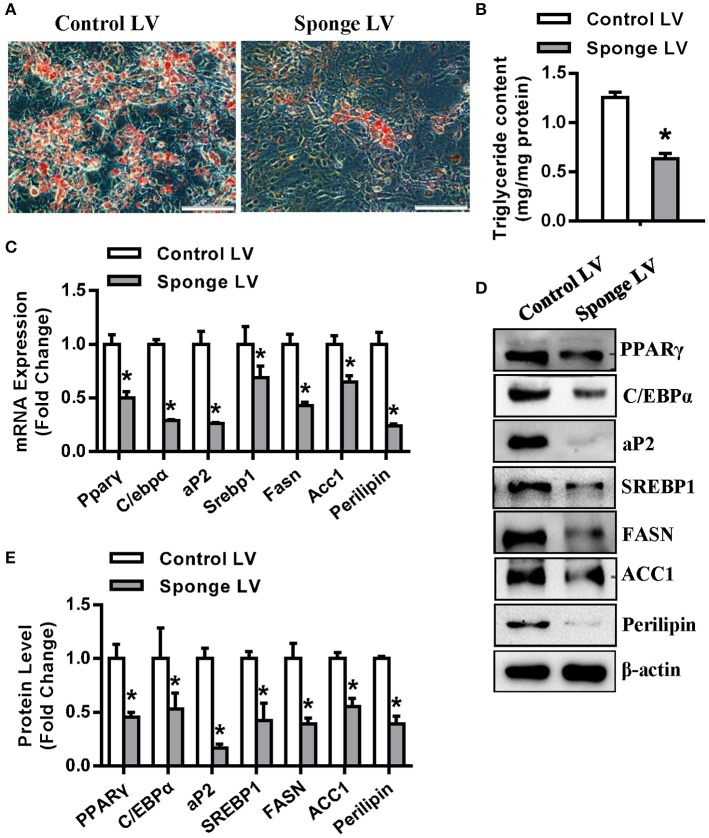
miR-20a-5p sponge lentivirus inhibited adipogenesis and lipogenesis. 3T3-L1 was infected with miR-20a-5p sponge lentivirus followed by adipogenic induction. **(A,B)** Differentiated adipocytes were stained with oil-red O and intracellular triglyceride contents were measured 5 days after adipogenic induction. The mRNA **(C)** and protein **(D)** levels of adipogenic and lipogenic factors were analyzed by qRT-PCR and Western blotting. **(E)** Protein expression levels were quantified. Scale in **(A)**: 200 μm. Values are the means ± SD (*n* = 3). *Significant *vs*. control LV, *p* < 0.05.

In addition, the miR-20a-5p sponge lentivirus (Sponge LV) were made according to previously published protocol (22). Oil-red O staining showed that the sponge LV infection resulted in fewer differentiated adipocytes and less triglyceride content as compared with control LV (Figures 4A,B). Gene expression assays revealed that miR-20a-5p Sponge LV inhibited the mRNA (Figure 4C) and protein (Figures 4D,E) expression of adipogenic and lipogenic factors in presence of adipogenic medium.

### Tob2 Was a Direct Target Gene of miR-20a-5p

**Figure 5:**
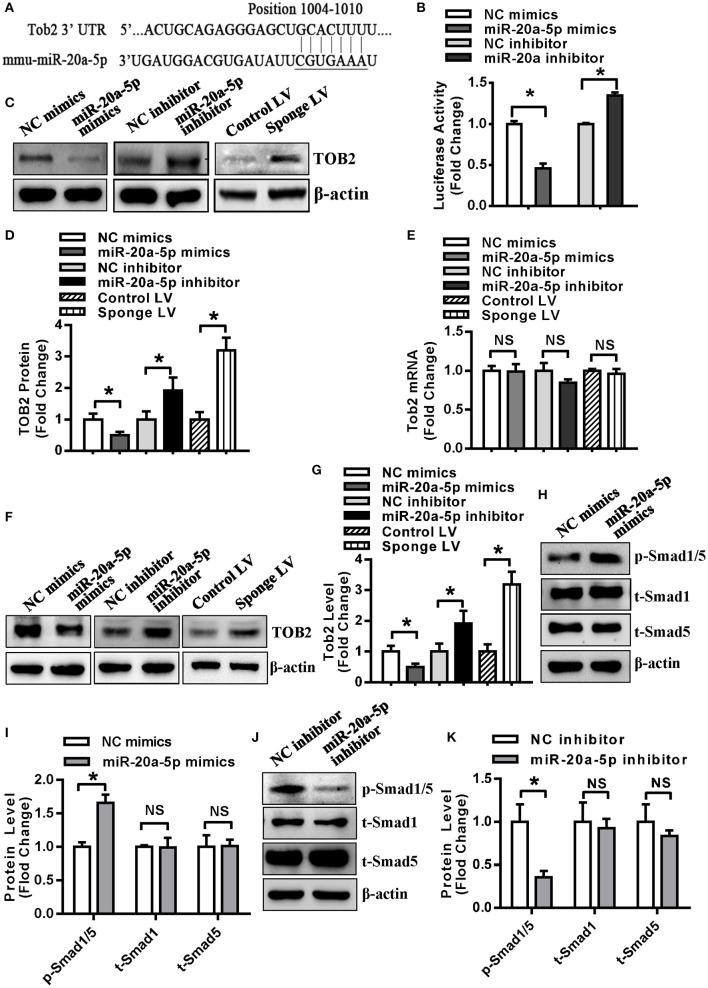
TOB2 was a direct target of miR-20a-5p. **(A)** Schematic representation of the alignment of Tob2 3'UTR and mir-20a-5p. **(B)** The activity of the Tob2 3′-UTR construct was detected in HEK-293 after co-transfection with miR-20a-5p mimics or inhibitor. The protein **(C,D)** and mRNA **(E)** levels of TOB2 were analyzed by Western blotting and qRT-PCR in undifferentiated 3T3-L1 transfected with miR-20a-5p mimics or inhibitor, or infected with sponge lentivirus. 3T3-L1 cells were allowed for 3 days of adipogenic differentiation, then treated with miR-20a-5p mimics or inhibitor, or sponge lentivirus for 2 days. The protein level of TOB2 was analyzed by Western blotting **(F)** and quantified **(G)**. Phosphorylated Smad1/5 protein was analyzed using Western blotting and quantified in undifferentiated 3T3-L1 transfected with miR-20a-5p mimics **(H,I)** or inhibitor **(J,K)**. Values are the means ± SD (*n* = 3). *Significant vs. NC, *p* < 0.05.

Kdm6b, Tgfbr2 and Klf3 have been demonstrated to be the direct targets of miR-20a-5p in our previous studies (21, 22). In this study, Tob2 was predicted to be a potential target of miR-20a-5p and the potential pairing between miR-20a-5p and Tob2 is shown in Figure 5A. The luciferase activity of Tob2 3′-UTR reporter construct was significantly decreased in HEK-293 cells supplemented with miR-20a-5p mimics, but increased in the cells transfected with miR-20a-5p inhibitor (Figure 5B). Furthermore, the protein level of TOB2 was reduced in undifferentiated 3T3-L1 supplemented with miR-20a-5p mimics, but induced in cells either transfected with miR-20a-5p inhibitor or infected with Sponge LV (Figures 5C,D). However, no significant change in the mRNA level of Tob2 was observed in the cells either transfected with miR-20a-5p mimics or inhibitor, or infected with Sponge LV (Figure 5E). These findings suggested that miR-20a-5p could regulate TOB2 expression via post-transcriptional repression. In differentiated 3T3-L1 cells after 3-day adipogenic treatment, transfection with miR-20a-5p mimics decreased TOB2 protein while miR-20a-5p inhibitor or Sponge LV increased TOB2 protein as well (Figures 5F,G), demonstrating the efficacy of transfection or infection in the differentiated cells.

It is reported that TOB2 inhibits PPARγ2 transcription by suppressing BMP2-induced Smad1/5 phosphorylation (25). We investigated the effect of miR-20a-5p on Smad1/5 phospharylation and the results showed that the level of phosphorylated Smad1/5 was increased in 3T3-L1 cells transfected with miR-20a-5p mimics while declined in cells transfected with miR-20a-5p inhibitor (Figures 5H–K).

**Figure 6:**
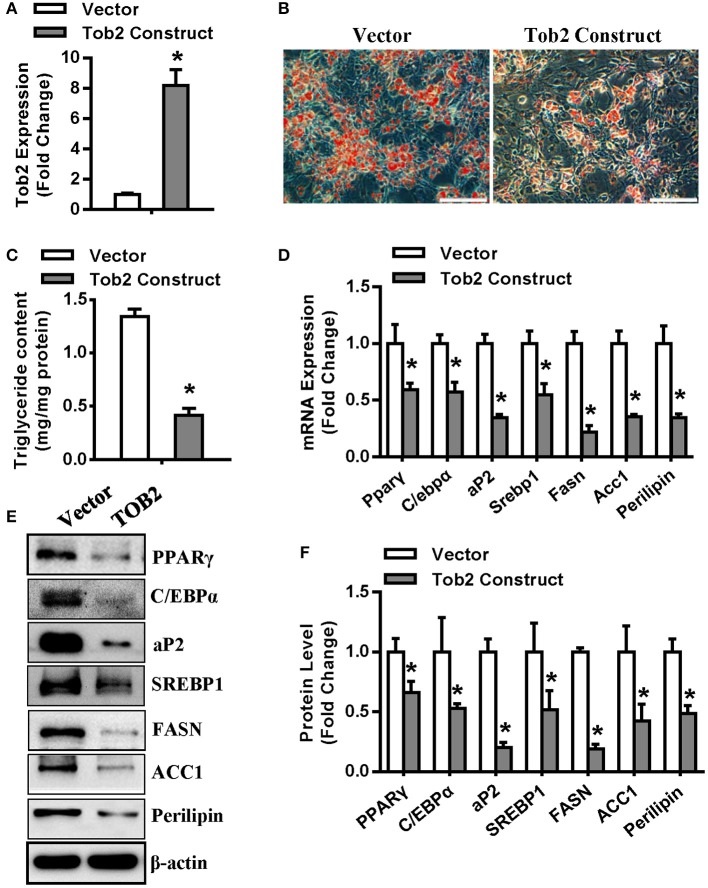
TOB2 inhibited adipocyte formation. **(A)** qRT-PCR verified the overexpression of Tob2 construct. **(B,C)** Tob2 overexpression inhibited adipocyte formation from 3T3-L1 cells after adipogenic treatment. The mRNA **(D)** and protein **(E,F)** levels of adipogenic factors and lipogenesis were analyzed. Scale in **(B)**: 200 μm. Values are the means ± SD (*n* = 3). *Significant vs. Vector, *p* < 0.05.

Then, experiments were conducted to further investigate the role of TOB2 in adipocyte differentiation. We first demonstrated the efficient overexpression of Tob2 in 3T3-L1 cells after transfection (Figure 6A). In presence of adipogenic agents, Tob2 overexpression restrained adipocyte formation and triglyceride storage (Figures 6B,C). The mRNA and protein levels of the adipogenic and lipogenic factors were decreased in the cells overexpressing TOB2 (Figures 6D–F). These results reveal the anti-adipogenic role of TOB2 in 3T3-L1 preadipocytes.

### Tob2 Overexpression Attenuated the Pro-Adipogenic and Pro-Lipogensic Effect of miR-20a-5p

**Figure 7:**
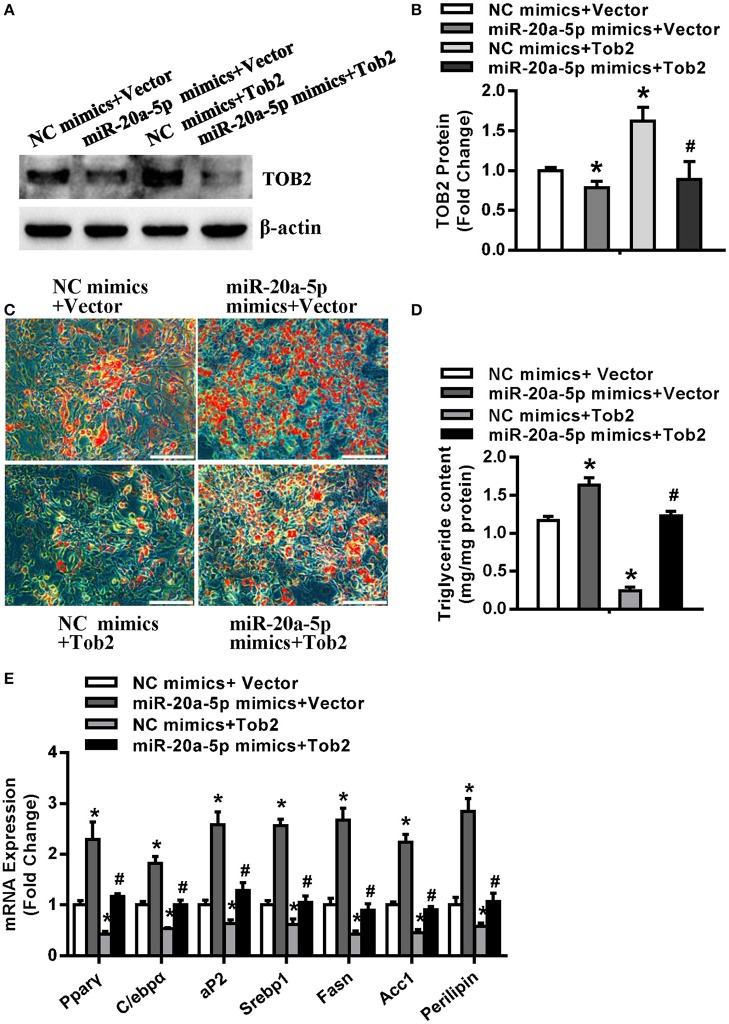
Overexpression of Tob2 attenuated miR-20a-5p stimulation of adipocyte differentiation and lipogenesis. **(A,B)** The expression of TOB2 was detected by Western blotting following cotransfection of miR-20a-5p or NC mimics and Tob2 construct or vector. **(C,D)** Oil-red O staining was done and triglyceride contents were measured. **(E)** The levels of adipogenic and lipogenic factors were analyzed by qRT-PCR. Scale in **(C)**: 200 μm. Values are the means ± SD (*n* = 3). *Significant vs. NC mimics plus Vector, *p* < 0.05; ^#^Significant vs. miR-20a-5p mimics plus Vector, *p* < 0.05.

Cells were cotransfected with miR-20a-5p or control mimics and Tob2 expression construct or vector. Western blotting experiment verified the increase of TOB2 in cells cotransfected with Tob2 construct. Supplementing miR-20a-5p reduced the protein level of TOB2 either in absence or presence of Tob2 construct (Figures 7A,B). The subsequent functional studies revealed that Tob2 overexpression significantly attenuated adipocyte formation and triglyceride storage induced by miR-20a-5p supplementation (Figures 7C,D). Consistently, the cotransfection of miR-20a-5p mimics and Tob2 construct significantly downregulated the mRNA expression of the adipogenic and lipogenic factors as compared to the cotransfection of miR-20a-5p mimics and vector (Figure 7E). The data suggest that the downregulation of TOB2 mediates the adipogenic and lipogenic role of miR-20a-5p.

### C/EBPα Regulated miR-20a-5p in 3T3-L1 Preadipocytes

**Figure 8:**
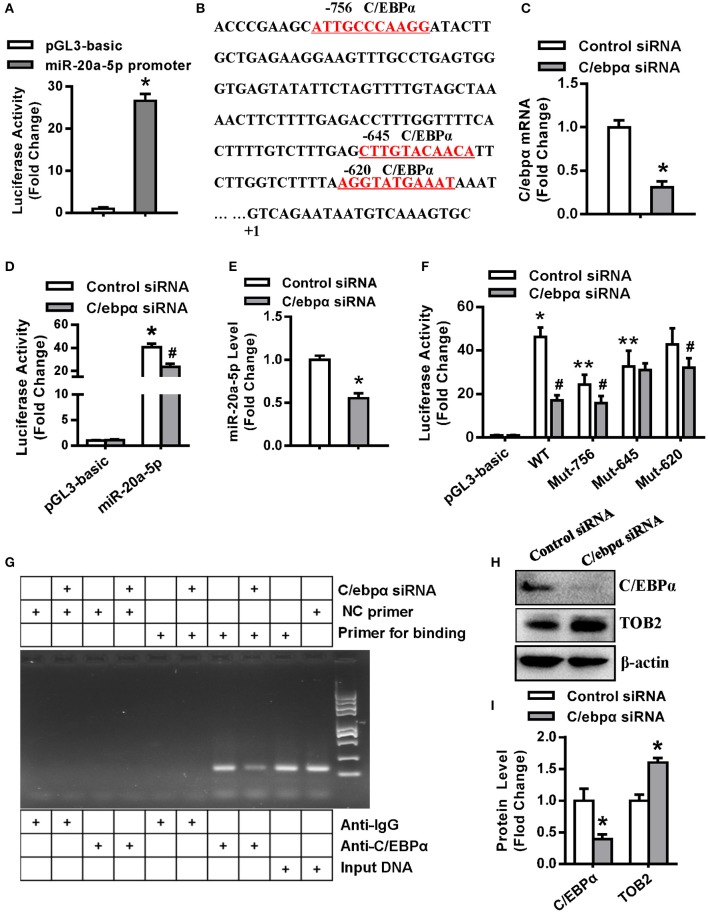
C/EBPα transcriptionally regulated miR-20a-5p expression. **(A)** The transcriptional activity of miR-20a-5p promoter in 3T3-L1 was assayed. **(B)** Putative C/EBPα binding sites (red and underlined) in miR-20a-5p promoter are shown. **(C)** The silencing efficacy of C/ebpα siRNA was verified by qRT-PCR. The transcriptional activity of miR-20a-5p promoter **(D)** in 3T3-L1 cotransfected with C/ebpα siRNA was assayed. **(E)** The expression level of miR-20a-5p was assayed in 3T3-L1 transfected with C/ebpα siRNA. **(F)** The wild-type or mutant construct of miR-20a-5p promoter was cotransfected with C/ebpα siRNA and luciferase activity was assayed. **(G)** PCR-based ChIP assay was done to detect the binding of C/EBPα to the miR-20a-5p promoter. **(H,I)** Effect of C/EBPα on TOB2 protein expression was analyzed and quantified. Values are means ± SD. **(A,D,F)**: *n* = 4, *Significant vs. pGL3-basic, *P* < 0.05, **Significant vs. WT promoter, *P* < 0.05; ^**#**^Significant vs. cotransfection with control siRNA, *P* < 0.05. **(C,E,I)**: *n* = 3, *Significant vs. control siRNA transfection, *p* < 0.05.

As shown in Figure 8A, the promoter construct of miR-20a-5p showed transcriptional activity in 3T3-L1 cells, with 26-fold increase of luciferase activity vs. the pGL3-basic vector (Figure 8A). The search of cis-acting elements revealed three putative C/EBPα binding motifs at −756, −645, and −620 nt, respectively (Figure 8B), suggesting that miR-20a-5p might be transcriptionally regulated by C/EBPα. We then studied if C/EBPα transactivates miR-20a-5p expression. The silencing efficacy of C/ebpα siRNA was demonstrated by using qRT-PCR (Figure 8C). Luciferase assay revealed that the depletion of C/EBPα significantly decreased the activity of the miR-20a-5p promoter (Figure 8D). Consistently, C/ebpα siRNA downregulated miR-20a-5p expression (Figure 8E). The point mutation of the C/EBPα binding motifs at either −756 or −645 nt attenuated the transcriptional activity of the promoter. By contrast, the mutation at −620 nt did not alter the promoter activity (Figure 8F). Furthermore, the cotransfection of C/ebpα siRNA did not further alter the promoter activity of the mutant construct with point mutation at −645, indicating the absence of other binding motifs within the construct. In contrast, the depletion of C/EBPα further decreased the activity of the mutant with mutation at −756 nt, suggesting the presence of other binding sites beyond this motif (Figure 8F). Taken together, the data suggest that the motif at −645 nt is the major response element for C/EBPα.

Then we examined the physical association of C/EBPα with the miR-20a-5p promoter by chromatin immunoprecipitation assay (ChIP) in 3T3-L1. The cells were transfected with either C/ebpα siRNA or control siRNA. PCR was done to amplify the promoter region harboring the C/EBPα binding site (−645 nt) using the DNA from the immunoprecipitates as the template. The expected fragment was successfully amplified in the samples incubated with anti-C/EBPα rather than with IgG. C/EBPα depletion attenuated the yield of the PCR product. By contrast, the expected fragment was not amplified in these samples using the negative control primers located far from the −645 nt region. These data demonstrate that C/EBPα physically bind the promoter at the −645 nt region (Figure 8G).

Moreover, we further assessed if C/EBPα affects TOB2 expression. The results showed that the depletion of C/EBPα induced TOB2 protein expression (Figures 8H,I).

## Discussion

In the current study, we found that the expression of miR-20a-5p was increased synchronously with C/EBPα in preadipocyte 3T3-L1 or precursor cells during adipogenesis. Moreover, miR-20a-5p level was upregulated in WAT of ob/ob mice and the mice fed with HFD. The data suggest miR-20a-5p may have an regulatory role in adipocyte differentiation.

We first tested the function of miR-20a-5p in adipogenesis and lipogenesis. Our data revealed that supplementing miR-20a-5p before adipogenic treatment promoted adipogenesis and lipogenesis of 3T3-L1 cells, as evidenced by the potentiated differentiation of adipocytes, excess triglyceride content, and upregulation of adipogenic and lipogenic factors. Conversely, inhibition of endogenous miR-20a-5p before the adipogenic induction suppressed adipogenesis and lipogenesis. Of note, in differentiated cells after 3 days of adipogenic treatment, either supplementation or inhibition of miR-20a-5p did not show any effect on the phenotypes of the mature adipocytes. These data suggest that miR-20a-5p promotes adipogenesis mainly at the early stage of differentiation.

We have recently reported that the miR-20a-5p sponge lentivirus inhibited adipogenesis of BMSCs (22). Consistently, in this research, we clarified that miR-20a-5p sponge LV had a negative effect on adipocyte differentiation as well as lipogenesis. These results suggest miR-20a-5p may be a novel target for the treatment of obesity and impaired lipid profiles.

miRNAs exert translational inhibition or degradation of the target mRNAs through binding to the complementary sites in the 3'-UTRs or the translated regions of the transcripts (10). To explore the mechanisms through which miR-20a-5p modulates adipogenesis and lipogenesis, the reporter construct of Tob2 3′-UTR was made that harbored the putative binding site of miR-20a-5p sequence. Luciferase assay showed that supplementing miR-20a-5p significantly suppressed, while inhibiting miR-20a-5p stimulated the luciferase activity of Tob2 3′-UTR reporter. Furthermore, supplementing miR-20a-5p in 3T3-L1 downregulated, while inhibiting miR-20a-5p upregulated TOB2 protein. These results confirmed that miR-20a-5p directly targets TOB2.

TOB2 is a member of TOB/BTG family, which consists of six antiproliferative proteins, i.e., TOB, TOB2, ANA/BTG3, BTG1, BTG2, and PC3B. TOB2 has recently been identified as a player in bone metabolism through suppressing both osteoclastogenesis (26) and osteogenesis (27). Moreover, TOB2 has regulatory effect on adipocyte. Takahashi et al. reported increased adiposity and increased expression of type 1A BMP receptor (BMPR1A) and PPARγ2 in Tob2 null mice. Accelerated adipogenesis was observed in primary tob2^−/−^ preadipocytes. TOB2 inhibits PPARγ2 expression by repressing BMP2-induced Smad1/5 phosphorylation and by sequestering C/EBPα from the promoter of Pparγ2 (26). In this research, we investigated the effect of miR-20a-5p on Smad1/5 phosphorylation and the data revealed the induction of phosphorylated Smad1/5 by miR-20a-5p. Thus, as the downstream signal of TOB2, Smad1/5 may also be implicated in the role of miR-20a-5p. In addition, we have previously reported that miR-20a-5p stimulated the expression of PPARγ in absence of adipogenic treatment, which might be partly due to the inhibitory effect of miR-20a-5p on TOB2 translation.

The current research revealed that miR-20a-5p at least partially lost its potential to stimulate adipocyte differentiation and lipogenesis under the background of Tob2 overexpression. In brief, Tob2 overexpression significantly attenuated adipocyte differentiation and triglyceride storage in cells induced by miR-20a-5p supplementation. The induced expression levels of the adipogenic and lipogenic factors after miR-20a-5p supplementation was suppressed when the cells was cotransfected with Tob2 construct. These results suggest that miR-20a-5p functions as a positive regulator of adipogenesis and lipogenesis through blocking TOB2 translation.

By now what interests us is the mechanism that controls transcriptional regulation of miR-20a-5p. To elucidate this, we made the promoter construct of miR-20a-5p and performed bioinformatics analysis. Several potential binding sites for C/EBPα were found within the promoter, three of which with highest scores are located at the regions −756, −645, and −620 nt. The results of luciferase assay showed that the knockdown of C/EBPα significantly decreased both the transcriptional activity of the wild-type miR-20a-5p promoter, and consistently, the expression level of miR-20a-5p. Further luciferase assays of the transfections with mutant promoter constructs under the background of C/ebpα silencing revealed the identity of the binding site at −645 nt as the response element of C/EBPα. Furthermore, ChIP experiment confirmed that C/EBPα physically binds this region within the miR-20a-5p promoter.

We thus develop a model that delineates how miR-20a-5p works. During adipogenesis and lipogenesis, the key transcription factor C/EBPα is expressed which then transcriptionally upregulates miR-20a-5p expression. In return, miR-20a-5p stimulates adipogenesis and lipogenesis via blocking the translation of TOB2, an inhibitor of adipogenesis and lipogenesis, which functions by inhibiting Smad1/5 phosphorylation and by suppressing C/EBPα recruitment to the PPARγ2 promoter. The upregulation of C/EBPα and PPARγ finally leads to adipocyte differentiation and lipogenesis (Figure S4). Thus, an novel regulatory circuit forms among C/EBPα, miR-20a-5p, and TOB2, which in concert regulates adipogenesis and lipogenesis.

## Conclusion

In summary, the present work has provided evidences that miR-20a-5p plays an important role in the regulation of adipogenesis and lipogenesis *in vitro*, which is based on a novel regulatory circuit of C/EBPα/miR-20a-5p/TOB2. Although many questions remain yet to be answered, our data suggest that serum miR-20a-5p might be a potential biomarker for obesity. It also suggests that fine-tuning miR-20a-5p level might be a beneficial therapeutic strategy for the treatment of obesity and related metabolic disorders.

## Data Availability Statement

The datasets generated for this study are available on request to the corresponding author.

## Ethics Statement

The animal study was reviewed and approved by Animal Ethics Committee of Tianjin Medical University.

## Author Contributions

JZ, JY, XW, ML, FL, EZ, and XuL performed the experiments. JZ analyzed the data and wrote the manuscript. XiL supervised the project. BW supervised and coordinated the project and refined the manuscript.

### Conflict of Interest

The authors declare that the research was conducted in the absence of any commercial or financial relationships that could be construed as a potential conflict of interest.

## References

[1] Kopelman PG. Obesity as a medical problem. Nature. (2000) 404:635–43. 10.1038/35007508 10766250

[2] Boonchaya-anant P Apovian CM Metabolically healthy obesity–does it exist? Curr Atheroscler Rep. (2014) 16:441 10.1007/s11883-014-0441-1 25092577

[3] Grundy SM. Obesity, metabolic syndrome, and cardiovascular disease. The J Clin Endocrinol Metab. (2004) 89:2595–600. 10.1210/jc.2004-0372 15181029

[4] Tontonoz P Hu E Spiegelman BM. Stimulation of adipogenesis in fibroblasts by PPAR gamma 2, a lipid-activated transcription factor. Cell. (1994) 79:1147–56. 10.1016/0092-8674(94)90006-X 8001151

[5] Zanotti S Stadmeyer L Smerdel-Ramoya A Durant D Canalis E. Misexpression of CCAAT/enhancer binding protein beta causes osteopenia. J Endocrinol. (2009) 201:263–74. 10.1677/JOE-08-0514 19218285 PMC2674520

[6] Kubota N Terauchi Y Miki H Tamemoto H Yamauchi T Komeda K. PPAR gamma mediates high-fat diet-induced adipocyte hypertrophy and insulin resistance. Mol Cell. (1999) 4:597–609. 10.1016/S1097-2765(00)80210-5 10549291

[7] Linhart HG Ishimura-Oka K DeMayo F Kibe T Repka D Poindexter B C/EBPalpha is required for differentiation of white, but not brown, adipose tissue. Proc Natl Acad Sci USA. (2001) 98:12532–7. 10.1073/pnas.211416898 11606718 PMC60088

[8] Evans RM Barish GD Wang YX. PPARs and the complex journey to obesity. Nat Med. (2004) 10:355–61. 10.1038/nm1025 15057233

[9] Gregoire FM. Adipocyte differentiation: from fibroblast to endocrine cell. Exp Biol Med. (2001) 226:997–1002. 10.1177/153537020122601106 11743135

[10] Bartel DP. MicroRNAs: genomics, biogenesis, mechanism, and function. Cell. (2004) 116:281–97. 10.1016/S0092-8674(04)00045-5 14744438

[11] Vienberg S Geiger J Madsen S Dalgaard LT. MicroRNAs in metabolism. Acta Physiol. (2017) 219:346–61. 10.1111/apha.12681 27009502 PMC5297868

[12] Ling HY Wen GB Feng SD Tuo QH Ou HS Yao CH. MicroRNA-375 promotes 3T3-L1 adipocyte differentiation through modulation of extracellular signal-regulated kinase signalling. Clin Exp Pharmacol Physiol. (2011) 38:239–46. 10.1111/j.1440-1681.2011.05493.x 21291493 PMC3086632

[13] Shi C Zhang M Tong M Yang L Pang L Chen L. miR-148a is associated with obesity and modulates adipocyte differentiation of mesenchymal stem cells through Wnt signaling. Sci Rep. (2015) 5:9930. 10.1038/srep09930 26001136 PMC4441322

[14] Chen H Wang S Chen L Chen Y Wu M Zhang Y. MicroRNA-344 inhibits 3T3-L1 cell differentiation via targeting GSK3beta of Wnt/beta-catenin signaling pathway. FEBS Lett. (2014) 588:429–35. 10.1016/j.febslet.2013.12.002 24333578

[15] Chen C Peng Y Peng J Jiang S. miR-135a-5p inhibits 3T3-L1 adipogenesis through activation of canonical Wnt/beta-catenin signaling. J Mol Endocrinol. (2014) 52:311–20. 10.1530/JME-14-0013 24850830

[16] Chen H Mo D Li M Zhang Y Chen L Zhang X. miR-709 inhibits 3T3-L1 cell differentiation by targeting GSK3beta of Wnt/beta-catenin signaling. Cell Signal. (2014) 26:2583–9. 10.1016/j.cellsig.2014.07.017 25038456

[17] Price NL Singh AK Rotllan N Goedeke L Wing A Canfran-Duque A. Genetic ablation of miR-33 increases food intake, enhances adipose tissue expansion, and promotes obesity and insulin resistance. Cell Rep. (2018) 22:2133–45. 10.1016/j.celrep.2018.01.074 29466739 PMC5860817

[18] Takanabe R Ono K Abe Y Takaya T Horie T Wada H. Up-regulated expression of microRNA-143 in association with obesity in adipose tissue of mice fed high-fat diet. Biochem Biophys Res Commun. (2008) 376:728–32. 10.1016/j.bbrc.2008.09.050 18809385

[19] Bai X Han G Liu Y Jiang H He Q. MiRNA-20a-5p promotes the growth of triple-negative breast cancer cells through targeting RUNX3. Biomed Pharmacother. (2018) 103:1482-9. 10.1016/j.biopha.2018.04.165 29864933

[20] Huang F Chen W Peng J Li Y Zhuang Y Zhu Z. LncRNA PVT1 triggers Cyto-protective autophagy and promotes pancreatic ductal adenocarcinoma development via the miR-20a-5p/ULK1 Axis. Mol Cancer. (2018) 17:98. 10.1186/s12943-018-0845-6 30001707 PMC6043995

[21] Zhou J Guo F Wang G Wang J Zheng F Guan X. miR-20a regulates adipocyte differentiation by targeting lysine-specific demethylase 6b and transforming growth factor-beta signaling. Int J Obes. (2015) 39:1282–91. 10.1038/ijo.2015.43 25817070

[22] Zhu E Zhang J Zhou J Yuan H Zhao W Wang B. miR-20a-5p promotes adipogenic differentiation of murine bone marrow stromal cells via targeting Kruppel-like factor 3. J Mol Endocrinol. (2018) 60:225–37. 10.1530/JME-17-0183 29348304

[23] Zhou J Wang S Qi Q Yang X Zhu E Yuan H. Nuclear factor I-C reciprocally regulates adipocyte and osteoblast differentiation via control of canonical Wnt signaling. FASEB J. (2017) 31:1939–52. 10.1096/fj.201600975RR 28122918

[24] Gao Y Li J Xu X Wang S Yang Y Zhou J. Embelin attenuates adipogenesis and lipogenesis through activating canonical Wnt signaling and inhibits high-fat diet-induced obesity. Int J Obes. (2017) 41:729–738. 10.1038/ijo.2017.35 28163317

[25] Takahashi A Morita M Yokoyama K Suzuki T Yamamoto T. Tob2 inhibits peroxisome proliferator-activated receptor gamma2 expression by sequestering Smads and C/EBPalpha during adipocyte differentiation. Mol Cell Biol. (2012) 32:5067–77. 10.1128/MCB.00610-12 23071089 PMC3510534

[26] Ajima R Akiyama T Usui M Yoneda M Yoshida Y Nakamura T. Osteoporotic bone formation in mice lacking tob2; involvement of Tob2 in RANK ligand expression and osteoclasts differentiation. FEBS Lett. (2008) 582:1313–8. 10.1016/j.febslet.2008.03.012 18358842

[27] Gamez B Rodriguez-Carballo E Bartrons R Rosa JL Ventura F. MicroRNA-322 (miR-322) and its target protein Tob2 modulate Osterix (Osx) mRNA stability. J Biol Chem. (2013) 288:14264–75. 10.1074/jbc.M112.432104 23564456 PMC3656283

