# Supplementary material for: A Novel Regulatory Circuit “C/EBPα/miR-20a-5p/TOB2” Regulates Adipogenesis and Lipogenesis
Source: Front Endocrinol (Lausanne). 2020 Jan 8;10:894. doi: 10.3389/fendo.2019.00894 (PMC6960138; doi:10.3389/fendo.2019.00894)
Supplement: Supplementary file 1 [file Table_1.DOCX]

**Table 1. Primers used for PCR amplifications**

| Gene Names | Forward primer sequences | Reverse primer sequences |
| --- | --- | --- |
| Pparγ | CTTGACAGGAAAGACAACGG | GCTTCTACGGATCGAAACTG |
| C/ebpα | CTGATTCTTGCCAAACTGAG | GAGGAAGCTAAGACCCACTAC |
| aP2 | AAATCACCGCAGACGACAGG | GGCTCATGCCCTTTCATAAAC |
| Srebp1 | CGACTACATCCGCTTCTTGCAG | CCTCCATAGACACATCTGTGCC |
| Fasn | CACAGTGCTCAAAGGACATGCC | CACCAGGTGTAGTGCCTTCCTC |
| Acc1 | GTTCTGTTGGACAACGCCTTCAC | GGAGTCACAGAAGCAGCCCATT |
| Perilipin | GAGAAGGTGGTAGAGTTCCTCC | GTGTGTCGAGAAAGAGTGTTGGC |
| Tob2 | GTGACAGTGAGGCGAGTGG | AAAGAGGCAGTAGTAAAGGTGATGG |
| β-actin | AAGACCTCTATGCCAACACAG | GGAGGAGCAATGATCTTGATC |
| C/EBPα ChIP | TGAGTGGGTGAGTATATTCTAG | CACTCATTTATGACAAGAGTTC |
| NC Primer | TGTGTCAGCTCCAATTTGG | GAGAGAAACACCACCACCC |
| Tob2 3’UTR | TGGGTCCTGGGTTCTATCAC | AGCTGAAGGCTCAGGACTGC |
| miR-20a promoter | ACTCGAGAATGAGCAACGTGCCACGAG | AAAGCTTGACTGGTCACAGCTTCAGTC |
| miR-20a-5p | ATGCTAAAGTGCTTATAGT | CAGTGCAGGGTCCGAGGTATTC |
| Full length Tob2 | TTGGTACCGAGCTCGGATCCGCCACC  ATGCAGCTGGAGATCAAAGTGGCC | TGCTGGATATCTGCAGAATTCTCAGTT  GGCCAGCACGACAG |
