# Supplementary material for: A Novel Regulatory Circuit “C/EBPα/miR-20a-5p/TOB2” Regulates Adipogenesis and Lipogenesis
Source: Front Endocrinol (Lausanne). 2020 Jan 8;10:894. doi: 10.3389/fendo.2019.00894 (PMC6960138; doi:10.3389/fendo.2019.00894)
Supplement: Supplementary file 2 [file Image_1.pdf]

Figure S1

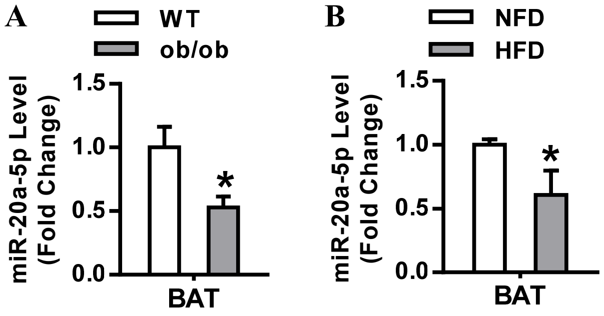

Figure S1. miR-20a-5p expression was reduced in brown adipose tissue of obese mice. qRT-PCR was done to examine the level of miR-20a-5p in interscapular brown adipose tissue of ob/ob (A) or HFD-fed (B) obese mice. Values are the means ± SD, n=5 in (A), n=3 in (B). *Significant *vs.* WT or NFD, $p < 0.05$.
