# Supplementary material for: A Novel Regulatory Circuit “C/EBPα/miR-20a-5p/TOB2” Regulates Adipogenesis and Lipogenesis
Source: Front Endocrinol (Lausanne). 2020 Jan 8;10:894. doi: 10.3389/fendo.2019.00894 (PMC6960138; doi:10.3389/fendo.2019.00894)
Supplement: Supplementary file 3 [file Image_2.pdf]

Figure S2

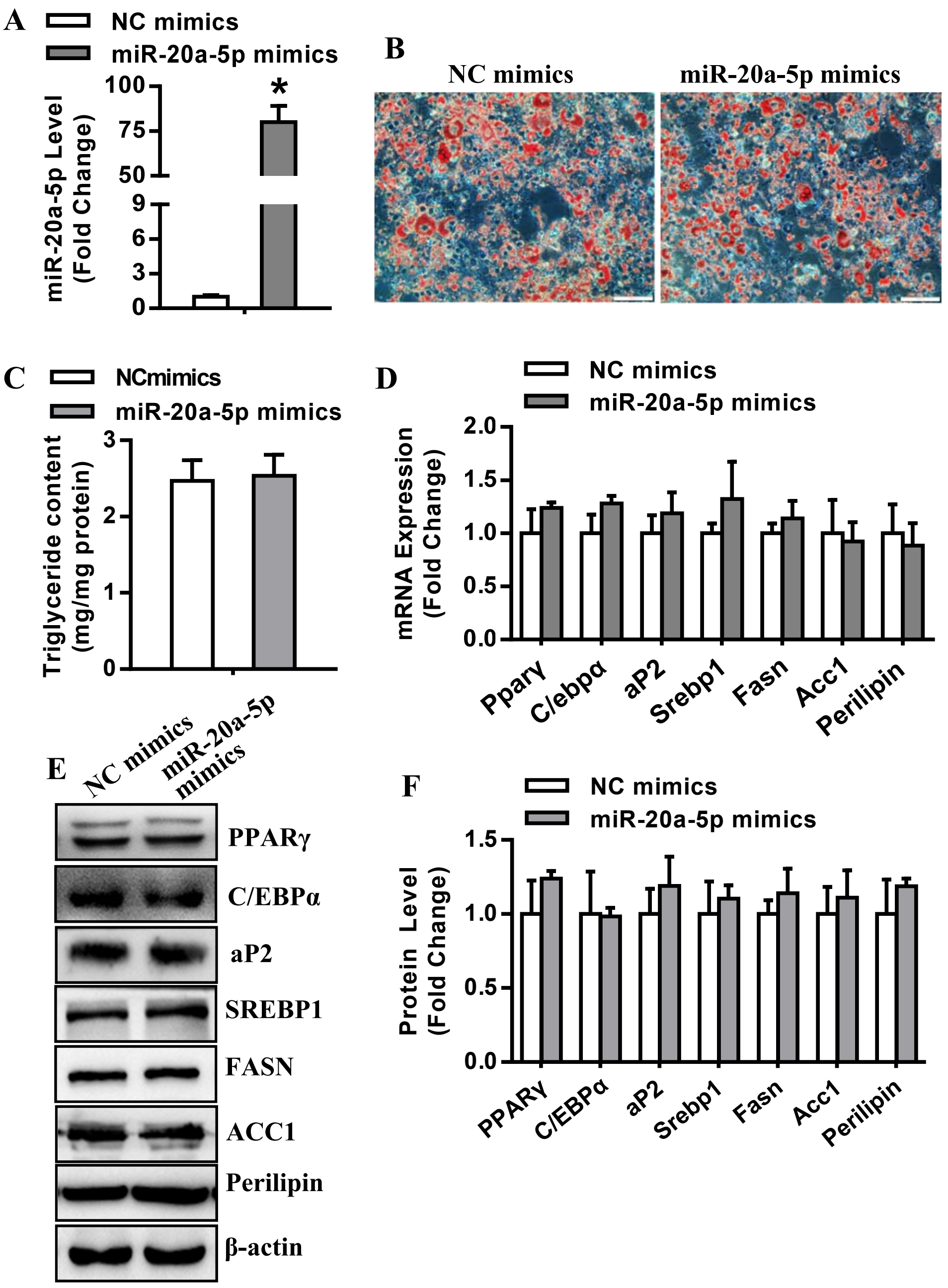

Figure S2. Supplementing miR-20a-5p in differentiated 3T3-L1 cells had no effect on mature adipocyte phenotypes. Transfection of miR-20a-5p or NC mimics was performed in differentiated 3T3-L1 under adipogenic treatment for 3 days. **(A)** qRT-PCR verified the increase of miR-20a-5p in differentiated 3T3-L1 2 days after transfection. **(B, C)** Differentiated adipocytes were stained with oil-red O and intracellular triglyceride contents were measured 7 days after transfection. The mRNA **(D)** and protein **(E, F)** levels of adipogenic and lipogenic factors were analyzed by qRT-PCR and Western blotting, respectively, 2 days after transfection. Scale in B: 200 μm. Values are the means ± SD (n=3). *Significant *vs*. NC, $p < 0.05$.
