# Supplementary material for: A Novel Regulatory Circuit “C/EBPα/miR-20a-5p/TOB2” Regulates Adipogenesis and Lipogenesis
Source: Front Endocrinol (Lausanne). 2020 Jan 8;10:894. doi: 10.3389/fendo.2019.00894 (PMC6960138; doi:10.3389/fendo.2019.00894)
Supplement: Supplementary file 4 [file Image_3.pdf]

Figure S3

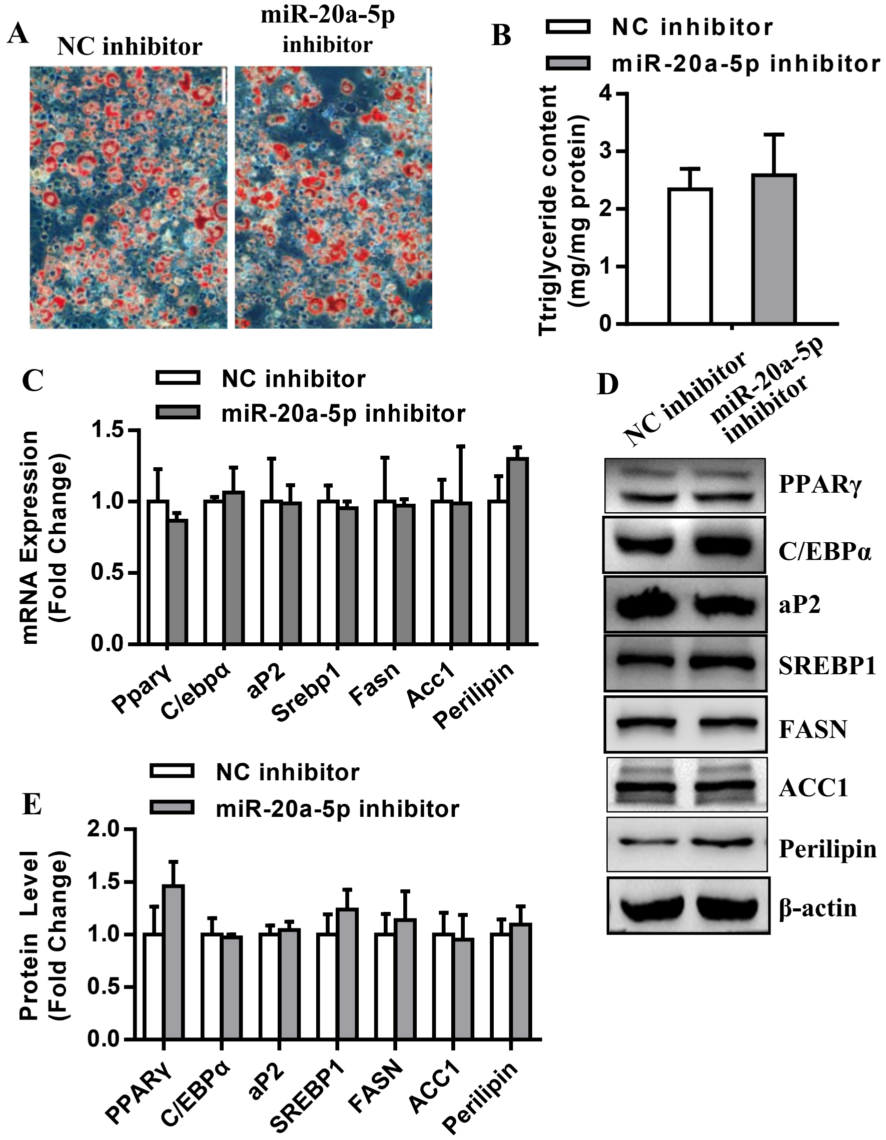

Figure S3. Inhibition of endogenous miR-20a-5p in differentiated 3T3-L1 had no effect on mature adipocyte phenotypes. Transfection of miR-20a-5p or NC inhibitor was performed in differentiated 3T3-L1 under adipogenic treatment for 3 days. **(A, B)** Differentiated adipocytes were stained with oil-red O and intracellular triglyceride contents were measured 7 days after transfection. The mRNA **(C)** and protein **(D, E)** levels of adipogenic and lipogenic factors were analyzed by qRT-PCR and Western blotting, respectively, 7 days after transfection. Scale in A: 200 μm. Values are means ± SD (n=3). *Significant vs. NC inhibitor, $p < 0.05$.
