# Supplementary material for: A Novel Regulatory Circuit “C/EBPα/miR-20a-5p/TOB2” Regulates Adipogenesis and Lipogenesis
Source: Front Endocrinol (Lausanne). 2020 Jan 8;10:894. doi: 10.3389/fendo.2019.00894 (PMC6960138; doi:10.3389/fendo.2019.00894)
Supplement: Supplementary file 5 [file Image_4.pdf]

Figure S4

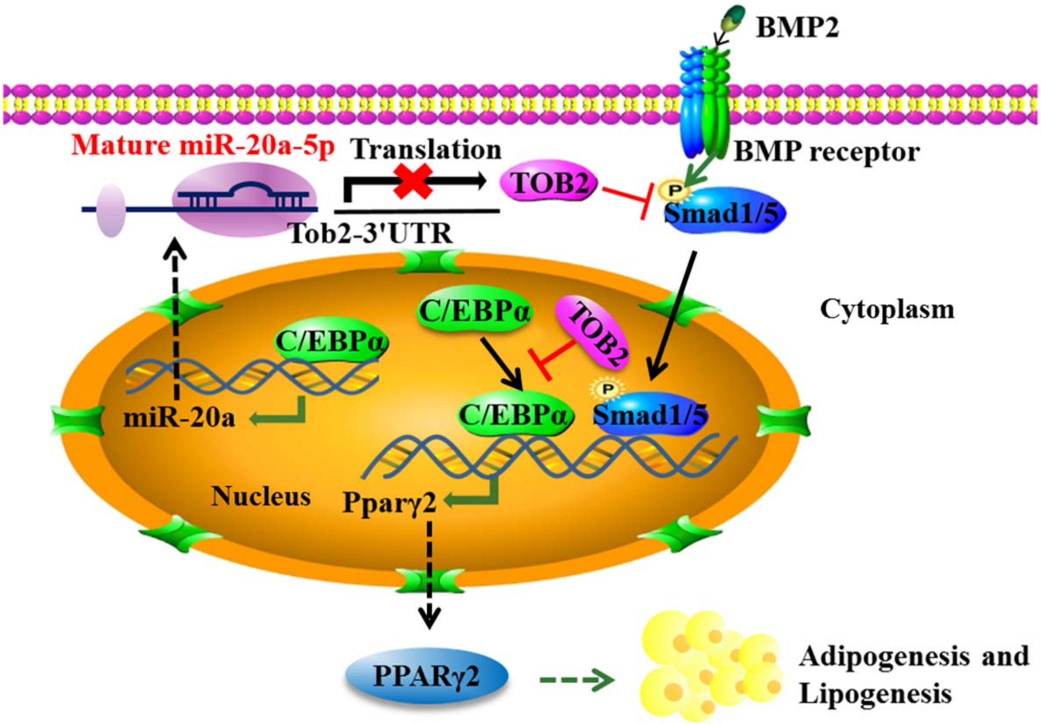

Figure S4. Schematic diagram depicting the mechanism for miR-20a-5p in regulating adipocyte differentiation.
